# Insecticide/acaricide resistance in fleas and ticks infesting dogs and cats

**DOI:** 10.1186/1756-3305-7-8

**Published:** 2014-01-06

**Authors:** Tad B Coles, Michael W Dryden

**Affiliations:** 1 Medical Writing and Veterinary Consulting, Overland Park, KS 66212, USA; 2 Department of Diagnostic Medicine and Pathobiology, Kansas State University, Manhattan, KS 66506, USA

**Keywords:** Flea, *Ctenocephalides felis*, Tick, *Rhipicephalus sanguineus*, Dog, Cat, Insecticide, Acaricide, Resistance, Refugia, Refugium

## Abstract

This review defines insecticide/acaricide resistance and describes the history, evolution, types, mechanisms, and detection of resistance as it applies to chemicals currently used against fleas and ticks of dogs and cats and summarizes resistance reported to date. We introduce the concept of refugia as it applies to flea and tick resistance and discuss strategies to minimize the impact and inevitable onset of resistance to newer classes of insecticides. Our purpose is to provide the veterinary practitioner with information needed to investigate suspected lack of efficacy, respond to lack of efficacy complaints from their clients, and evaluate the relative importance of resistance as they strive to relieve their patients and satisfy their clients when faced with flea and tick infestations that are difficult to resolve. We conclude that causality of suspected lack of insecticide/acaricide efficacy is most likely treatment deficiency, not resistance.

## Review

### Background

In this paper we will review the current information relative to resistance of fleas and ticks to insecticides and acaricides, respectively, as it applies to canine and feline veterinary practitioners. Veterinarians must provide answers to pet owners with unmet expectations and there are many reasons clients voice dissatisfaction. Investigating possible inconsistency regarding insecticide/acaricide treatment of all pet mammals in the household and determining if neighboring pets or flea-infested wildlife might be serving as a source of reinfestation is imperative and will often point to obvious strategies to improve efficacy and client satisfaction [1]. Clients often bring up resistance to insecticides/acaricides as soon as they see evidence of fleas or ticks on their recently treated pet. The following general review of insecticide/acaricide resistance, focusing on species of fleas and ticks that infest dogs and cats, will help veterinarians respond to client concerns.

Some 2,500 flea species have been described, at least 15 of which occasionally infest dogs and cats [2]. However, only a few flea species are significant disease-carrying and nuisance pests of dogs, cats, and their human owners: *Ctenocephalides felis felis* (cat flea), *C. canis* (dog flea), *Echidnophaga gallinacea* (sticktight flea), *Pulex irritans* (human flea), and the closely related *P. simulans*[2-4]. *Ctenocephalides felis* is by far the most common flea infesting dogs and cats around the world [2,4,5]. In one study, all of the 972 flea field isolates obtained from dogs and cats from 2001 to 2005 in the United States, the United Kingdom, and Germany were *Ctenocephalides felis*[6].

Dogs and cats reportedly serve as bridging hosts for a variety of flea species, acquiring fleas from wild animals and bringing them home to infest other domestic animals and pester people [2], but it is more likely that dogs and cats serve as an original flea source in that they carry *Ctenocephalides felis* to urban wild animals, which serve as reservoir hosts maintaining a flea population that reinfests pet dogs and cats after treatment.

Dogs in North America are most commonly infested with the following tick species: *Amblyomma americanum* (Lone Star tick), *A. maculatum* (Gulf Coast tick), *Dermacentor variabilis* (American dog tick), *D. andersoni* (Rocky Mountain wood tick), *D. occidentalis* (Pacific Coast tick), *Ixodes pacificus* (western black-legged tick), *I. scapularis* (black-legged tick), *Otobius megnini* (spinose ear tick), and *Rhipicephalus sanguineus* (brown dog tick) [5,7]. Cats, while not as commonly infested as dogs, are parasitized by *A. americanum, D. variabilis,* and *I. scapularis*[7].

As a point of clarification, fleas and ticks are arthropods, but of these two, only fleas are insects and, as such, we use insecticides to kill them. Ticks are not insects, but are arachnids (class arachnida as are mites and spiders) and, as such, we use acaricides to kill them. Different compounds have varying degrees of insecticidal and/or acaricidal properties.

### History and definition of resistance

The first report of insecticide/acaricide resistance was not in fleas or ticks. Melander raised the general insecticide-resistance topic a century ago in 1914 when he wondered if fruit-tree pest insects could become resistant to insecticide spray [8]. His answer to the query, “Can insects become resistant to sprays?” was his discovery that certain populations of San Jose scale insects at certain locales were still alive after being sprayed with sulphur-lime concentrations that killed all scale insects at other locales, a report widely referenced as the first documented evidence of insecticide resistance. But, while this is often cited as evidence of resistance, what he actually proved was that different scale insect populations separated by locale had different susceptibilities or tolerance to this insecticide. Whether or not the differences were due to acquired genetic resistance is unknown.

While resistance and tolerance are often used interchangeably, they are not the same. In contrast to resistance, tolerance is a natural tendency rather than a result of selection pressure [9]. Certain individuals are more tolerant of a specific pesticide dose than others. Sometimes it is difficult to differentiate true resistance from the natural range of pesticide susceptibility that exists as a bell curve in every population of pests [10]. Tolerance is also used to describe natural differences between different species or between life stages of organisms [11]. For example ticks are naturally more tolerant of imidacloprid than fleas and *Trichuris vulpis* is more tolerant of pyrantel pamoate than is *Ancylostoma caninum.*

What constitutes proof of resistance and how is resistance defined? The definition of resistance has changed with time. The World Health Organization (WHO) has served as the global coordinator for information on vector resistance and on standardization of pesticide-resistance measurements by providing methodology and test kits used to measure resistance. In 1957 the WHO [12] defined resistance as, “the development of an ability to tolerate toxicants which would prove lethal to the majority of individuals in a normal population of the same species.” Later, in 1992, the WHO [13] defined resistance in arthropods as, “an inherited characteristic that imparts an increased tolerance to a pesticide, or group of pesticides, such that the resistant individuals survive a concentration of the compound(s) that would normally be lethal to the species.” Even this latter definition is problematic because it includes the term “tolerance.”

Scientific literature is full of different definitions of “resistance,” which should be kept in mind as historical reports of “resistance” are reviewed. After Melander introduced the topic of resistance, the most frequently reported research on insecticide resistance has concerned crop pests and insect vectors of human disease, especially mosquitoes. Mosquitoes began to show resistance to DDT at about the same time that housefly resistance to DDT was first noted in Italy in 1946 [14]. Flea resistance was first noted in 1949 in Peruvian *Pulex irritans* that were resistant to dichlorodiphenyltrichloroethane (DDT) [15]. *Ctenocephalides felis* resistance to DDT was first reported in 1952 followed by reports of resistance to benzene hexachloride (BHC) and dieldrin in 1956 [16]. Tick resistance was first noted in 1954 to dieldrin in *Rhipicephalus sanguineus*[15]. Suspected resistance of *Dermacentor variabilis* to DDT, BHC, and dieldrin was reported in 1959 [16]. The number of arthropod species with suspected insecticide/acaricide resistance increased to 37 in 1955, with “inescapable and quantitative proof” of resistance in 18 of those species [15].

For this paper, our definition of insecticide/acaricide resistance is the selection of a specific heritable trait (or traits) in a population of arthropods, due to that population’s contact with a chemical, that results in a significant increase in the percentage of the population that will survive a standard dose of that chemical (or a closely related chemical in the case of cross resistance).

### Evolution of resistance

Individuals with genetic traits that allow them to survive exposure to an insecticide/acaricide will pass genes on to the subsequent generation, thereby potentially increasing the percentage of a population that can survive subsequent exposure to the chemical [1]. Within this more restricted definition of insecticide/acaricide resistance, the inherent bell curve based susceptibility differences in a “normal” population should be remembered [17], because the susceptibility of the new population is compared with the old or “normal” population when looking for a significant increase in survivability. There are three necessary conditions for evolution of resistance to occur:

1. Individuals in the population must differ genetically

2. Genetic differences must produce a phenotypic difference

3. The phenotypic difference must enhance survivability, transferring the resistance to the next generation [17]

Resistance genes develop through natural processes such as mutation and recombination. Continued use of parasiticides that kill arthropods lacking resistance genes selects for individuals with resistance genes. Therefore, insecticide or acaricide resistance is essentially time-compressed evolution. Parasiticides do not cause resistance per se; they contribute to the process by allowing survival of resistant individuals [6]. Melander wondered if the difference in insecticide susceptibility that he saw between populations of scale insects was a result of acclimatization or immunity, after ingesting small amounts of insecticide over a period of time, or if they had developed an actual hereditary resistance. If Melander had demonstrated an actual hereditary difference between populations was responsible for the change in susceptibility or if he had shown that susceptibility differences of an insect population had changed over time, then he would have documented resistance as defined herein.

### Types and mechanisms of resistance

In 2012 the WHO expanded their insecticide resistance definition by including three types of resistance [18]. They introduced these types by explaining that resistance referred to an evolutionary phenomenon whereby an insect was no longer killed by the standard dose of insecticide. These are the three types of resistance, or ways of looking at resistance, that the WHO identified:

● Molecular genotyping of resistance – Identification of the underlying genes that confer the inherited resistance trait, which provides evidence of the evolutionary process.

● Phenotypic resistance – Measurement of susceptibility when subjected to a standard dose, referring back to their 1957 definition of resistance as “development of an ability, in a strain of insects, to tolerate doses of toxicants, which would prove lethal to the majority of individuals in a normal population of the same species.”

● Resistance leading to control failure – Referring to an insecticide’s failure to control an insect vector’s transmission of disease, the WHO was primarily concerned with malaria. This “control failure” could be considered as a failure to control dermatitis caused by fleas or failure to control the various flea- and tick-transmitted diseases.

In addition four mechanisms of resistance have been identified: [17,18]

● Target site sensitivity

● Metabolic

● Behavioral

● Cuticular or reduced penetration

Target site sensitivity refers to induction of resistance via alteration of target site neuronal enzymes and receptors such that the insecticide/acaricide no longer binds effectively, hence the flea or tick is unaffected. As an example, organophosphate and carbamate insecticides inhibit acetylcholinesterase (AChE). Arthropod populations become resistant to these compounds when individuals within the population develop a structurally modified AChE enzyme that allows them to survive exposure to organophosphate and carbamate insecticides that kill the susceptible individuals within the population.

Metabolic resistance relies upon a) alteration of enzyme systems that arthropods use to detoxify foreign materials or b) prevention of the insecticide/acaricide from reaching its site of action. This occurs with esterases, oxidases, oxygenases, hydrolases, and glutathione-s transferases [17,18].

The latter two types of resistance (behavioral and cuticular) are not as common as the first two and are considered less important. Behaviorally resistant insects have behaviors that reduce contact with the insecticide, such as an increased tendency to move away from a treated surface or area. It is often difficult to assess if behavioral avoidance is genetic or adaptive [17,18]. Reduced cuticular penetration slows the uptake of an insecticide. This is not typically very effective unless combined with other mechanisms of resistance [17].

Study of insecticide/acaricide resistance typically occurs in the following sequence:

1. Resistance detected in a population

2. Individual arthropods collected and colonized in the lab

3. Colony is subjected to insecticidal/acaricidal selection pressure to increase the frequency of resistant individuals

4. Genetic control of resistance is characterized

5. Characterization of mechanism(s) of resistance [17]

### Problems related to detection and/or reports of resistance in clinical settings

How is resistance detected? While it might seem that resistance of fleas and ticks would become readily apparent to veterinarians because of increased pet owner complaints of continued observance of fleas and ticks in the face of treatment or evidence of flea or tick transmitted diseases, this is not typically the case. It can be difficult, if not impossible at times, for practitioners to differentiate between parasite resistance and other causes of inefficacy due to a multitude of environmental, host, and client variables. First, inconsistencies in client compliance must be considered [19]. Second, particularly with fleas, how long have insecticide treatments been ongoing? This is important given the well-known 2 to 3 month flea emergence pattern that occurs following initiation of topical and systemic treatments [1]. Flea eggs deposited in the premises before treatment will continue to develop and newly emergent fleas will continue to populate the home for at least a couple of months posttreatment, regardless of the type of pet treatment [1]. Depending on the number of eggs and rate of larval survivability, the problem may very well get worse before it improves [1]. In addition, seasonal and annual fluctuations in flea and tick populations caused by environmental changes or an influx of wildlife serving as reservoir hosts, can dramatically influence infestation pressure [5,6] and apparent treatment response. Finally, natural variations in the susceptibilities of different flea and tick populations can certainly impact control programs. Even though practitioners may suspect resistance and may have even encountered true resistance, given all these potential factors affecting control, case reports of individual failures cannot be construed as documenting resistance.

Does monitoring incidence or prevalence of flea-caused, flea-borne, and tick-borne diseases provide an accurate reflection of insecticide/acaricide resistance? Flea infestations of pets are associated with flea allergy dermatitis, iron deficiency anemia, and tapeworms (*Dipylidium caninum*) in dogs and cats; plague (caused by *Yersinia pestis*) in cats; bartonellosis (caused by Bartonella spp.) in dogs, cats and humans; and murine typhus (caused by *Rickettsia typhi or R. felis*) in humans [2,4]. Tick-borne diseases include *Anaplasma platys, A. phagocytophilum, Borrelia burgdorferi, Babesia canis, B. gibsoni, B. microti, Borrelia lonestari, Cytauxzoon felis, Ehrlichia canis, E. chaffeensis, E. ewingii, Francisella tularensis, Hepatozoon americanum, Rickettsia rickettsii,* and tick paralysis [7]. The relationship between insecticide resistance of mosquitoes and vector-borne diseases has been studied more extensively than that of fleas and ticks. While it would make sense that increased insecticide resistance of vectors could lead to decreased control of vector-borne diseases, this is not necessarily the case. Some insecticide-resistant mosquitoes have reduced fitness, a shorter lifespan, or carry lower burdens of filarial parasites, which can decrease incidence of vector-borne diseases while the population of insecticide-resistant mosquitoes rises [20]. On the other hand, increases in tick population unrelated to resistance can be associated with increased incidence of tick-borne diseases [4]. The bottom line is that the effect of insecticide/acaricide resistant flea and tick populations on the risk of flea- and tick-borne disease is unknown. Thus monitoring for increased incidence or prevalence of flea-caused, flea-borne, and tick-borne diseases may not be a reliable method for detecting arthropod resistance.

### Detection of resistance in the laboratory

In contrast, surveying flea and tick populations and using bioassays to compare susceptibility between populations is a far more reliable approach to determining resistance. WHO test kits have been used for many years to both detect and monitor flea and tick susceptibility [13,21]. The WHO filter paper method and various modifications used to screen for flea susceptibility to various insecticides is discussed by Moyses [10]. A topical application bioassay has been used to compare insecticide activity against fleas [22]. In addition, a flea larval bioassay was developed to monitor for susceptibility to imidacloprid [23]. However, while this assay has been used to evaluate dozens of isolates, the capability of larval susceptibility to predict subsequent adult flea susceptibility or resistance has not been established.

For ticks, in addition to the WHO test kits, the Food and Agriculture Organization (FAO) Larval Packet Test (LPT) is a standard bioassay used to measure tick susceptibility to acaricides [24]. The FAO-LPT involves placing tick larvae in a paper packet treated with a known quantity of acaricide [24-26]. Numerous other bioassay systems have been devised including larval and adult immersion tests [26-28].

A tick larval immersion microassay (LIM) was developed and LIM drug potency benchmarks for organophosphate, pyrethroid, carbamate, formamidine, macrocyclic lactones, and pyrazole acaricides have been established for the following ticks of importance to dogs and cats: *Amblyomma americanum* (Lone Star tick), *A. maculatum* (Gulf Coast tick), *Dermacentor variabilis* (American dog tick), and *Rhipicephalus sanguineus* (brown dog tick) [27]. In addition, a larval tarsal test has been developed involving placement of tick eggs into multi-well plates to allow the evaluation of multiple chemicals [29,30].

Another method of evaluating differences in susceptibility (and potentially resistance) is to administer test compounds directly to animals infested with different flea or tick populations and compare subsequent flea or tick counts, flea egg counts, and flea egg viability in negative controls and treated groups of animals [31]. Such evaluations can demonstrate differences in susceptibility between populations and provide data that are more directly applicable to veterinary practitioners; however, these studies are expensive and time consuming and have not been commonly used.

If genetic mutations are associated with insecticide or acaricide resistance, then testing for genetic mutation frequency in a flea or tick population can indirectly measure for the level of resistance in that population. Polymerase Chain Reaction (PCR) assays have been developed to test individual fleas for the presence of gene mutations associated with resistance to pyrethroids, the common knockdown resistance (kdr) mutation and super-kdr mutations [32].

Monitoring for emerging resistance by searching for a new mutation is difficult. As part of a program to proactively monitor cat flea populations for reduced susceptibility to imidacloprid prior to the onset of resistance, seven genes were identified that encode for cat flea nicotinic acetylcholine receptors (the receptor by which imidacloprid elicits its insecticidal affect) [33]. Monitoring fleas prior to their development of resistance is prudent because imidacloprid is commonly used against insect species other than fleas, e.g., aphids and whiteflies, and because brown planthoppers (*Nilaparvata lugens*) have shown target-site resistance to imidacloprid [33]. This genetic knowledge base will speed the development of PCR assays to detect emerging resistance in flea populations if they develop a new mutation for imidacloprid resistance.

A PCR assay was developed to test individual fleas for the “*Resistance to dieldrin*” or *Rdl* gene [34,35]. The *Rdl* gene is associated with cross-resistance to fipronil in other insect species, but has not yet been proven to be associated with flea resistance to currently used insecticides [36]. However, results of two studies that identified flea strains with reduced susceptibility to fipronil might suggest that some flea strains may be fipronil resistant (discussed in more depth later) [31,37].

One issue that is often raised when discussing resistance is how long to wait to reintroduce an insecticide after resistance has caused control problems. There is no easy answer to that question. For example, dieldrin has not been used as a pesticide since the 1980s. Lack of dieldrin use and the corresponding reduction in selection pressure would be expected to decrease the prevalence of these resistance genes; however the *Rdl* gene still persists in insect genomes [36]. Persistence of genetic resistance varies with different chemicals. The *Rdl* gene persists in many insect species (mosquitoes, gnats, houseflies) despite discontinued use of this pesticide [38]. Conversely, insect resistance to DDT and organophosphates showed rapid reversion upon cessation of use and decreased selection pressure [38]. Decreased *Ctenocephalides felis* resistance toward organophosphates (chlorpyrifos and malathion) was noted one year after organophosphate selection pressure was removed [39].

Another way to monitor for emerging resistance is to check for heritable alteration in enzyme systems arthropods use to detoxify foreign materials or prevent a chemical from reaching its site of action. One example of this detoxification mechanism is that increased esterase activity in insects negates the effects of pyrethroid and other classes of insecticides. The development of an assay to evaluate fleas for elevated esterase [40] improved the ability to make resistance management decisions because its use can provide a preliminary indication of resistance by estimating the frequency of resistance alleles in a population. This process can provide an earlier warning sign of emerging resistance than other methods such as resistance ratio (RR) determination. The RR is the ratio of the lethal dose in tested strain to that of a susceptible reference strain.

### Reports of resistance

*Ctenocephalides felis* resistance has been reported to: carbamates, organophosphates, pyrethroids, pyrethrins, organochlorines, and fipronil - more categories than any other flea species [13,37,41,42]. A flea strain from Florida was found to have RRs of 6.8 to cyfluthrin, 5.2 to cypermethrin, and 4.8 to fluvalinate, compared with a flea strain from California [43]. Regarding chemicals currently in use in the United States against fleas, *Ctenocephalides felis* resistance has been found for permethrin at an RR of 12 [10], chlorpyrifos at an RR of 10 [44], and propoxur at an RR of 4.4 [44,45]. *Ctenocephalides felis* resistance to fipronil was reported in a field strain collected from an efficacy complaint case, which had an RR of 26 for the LD_50_ (Lethal Dose – that killed 50% of the treated population) and an RR of 25 for the LD_95_ when compared with a fipronil-susceptible strain selected by industry-competitor scientists [37]. No cross-resistance to nitenpyram was found in the fipronil-resistant strain [37], which is not unexpected because the two compounds have different modes of action.

While RRs are often used in laboratory assays to evaluate susceptibility differences between insect strains, very little data exists to ascertain what those RRs actually mean to veterinary practitioners trying to eliminate a flea infestation. One study did look at RRs and corresponding efficacy of fipronil against fleas on cats [31]. That study compared fipronil susceptibility of two laboratory flea strains colonized prior to commercial introduction of fipronil with a Florida field strain and found that, while fipronil was ≥ 99.5% effective against adults of all three strains on the first day of treatment, the residual activity of fipronil against the field strain was significantly reduced. The RR of the field strain as compared to the most susceptible laboratory strain was only 2.1, but that low RR dropped the 30-day residual efficacy of fipronil from 100% to 77.3% [31]. This illustrates that a large change in residual efficacy may be associated with a relatively small RR change. Additionally, when an RR is reported between two populations, it does not necessarily mean that one population is resistant (as defined in this paper); it may simply mean that the assay detected naturally occurring differences in susceptibility between the populations.

El-Gazzar *et al.* suspected resistance when they found that a Florida flea strain was more tolerant than a California strain to nine insecticides (bendiocarb, carbaryl, propoxur, chlorpyrifos, malathion, chlorfenvinphos, diazinon, isofenphos, and propetamphos) [44]. After housing this strain in the laboratory for a year, during which cats used in flea production were occasionally treated with 5% carbaryl dust to reduce irritation and hair loss, researchers found this colony of fleas had increased resistance toward carbamates (bendiocarb, carbaryl, and propoxur), decreased resistance toward organophosphates (chlorpyrifos and malathion), and unchanged resistance toward chlorfenvinphos, diazinon, isofenphos, and propetamphos [39]. They suspected that colony exposure to carbaryl induced increased resistance toward carbamates [39].

A laboratory assay capable of monitoring susceptibility of *Ctenocephalides felis* to imidacloprid [23,44,46], was used to find populations with decreased susceptibility, which were then retested at a diagnostic dose of 3 ppm to evaluate for resistance [6,47]. Field strains of fleas with >5% adult emergence after exposure to imidacloprid treatment (6 such strains were reported in 2006 and 22 strains in 2011) were further investigated; however, none of these isolates were classified by the bioassay as resistant to imidacloprid [6,47].

The KS1 strain of *Ctenocephalides felis*, which was collected from dogs and cats in a Kansas shelter in 1990 and has since been maintained in a laboratory, has documented resistance or natural reduced susceptibility to carbaryl, chlorpyrifos, fenthion, fipronil, imidacloprid, permethrin, pyrethrins, and spinosad [23,31,32,48-52]. Based on bioassay and genetic analysis the cause of reduced efficacy of pyrethroid- and organophosphate-based products with this strain is likely true resistance [32,48,49]. However, insecticides such as fipronil, imidacloprid, and spinosad, which also have reduced activity against the KS1 strain [23,31,50-52] were commercially introduced into the United States market 6 years (fipronil and imidacloprid) or 17 years (spinosad) after the KS1 strain was colonized. The 28–30 day residual activity of fipronil, imidacloprid, and spinosad ranges from 95% to 100% with other flea strains, but is markedly reduced when tested against the KS1 strain [31,50,53,54]. In contrast, other recently introduced and currently used residual insecticides (indoxacarb, dinotefuran, and selamectin) have excellent residual activity against KS1 strain fleas [50-52,55].

The KS1 flea strain was isolated with no exposure to newer insecticides and no introduction of fleas from outside the colony. Could the KS1 strain have developed resistance to fipronil, imidacloprid, and spinosad? Does KS1 have an innate reduced susceptibility? Is the lack of efficacy due to prior KS1 selection associated with a different chemical that imparted cross-resistance to these chemicals?

According to Reinemeyer and Nielsen [56], a fellow parasitologist is fond of saying, “Somewhere in the world, worms exist that are resistant to a class of drugs that hasn’t been discovered yet.” But are such parasites truly resistant as we define the term, tolerant, or do they simply have a naturally reduced susceptibility? If the parasite population has not yet been exposed to the parasiticide (or a closely related parasiticide) and has not evolved (through selection) to survive exposure, then that population cannot be defined as resistant. Even if the drug is not lethal to the population and even if a higher percent of the population than expected survives parasiticide exposure, that population is not by definition resistant. The cause of decreased efficacy may be tolerance if there are differences in susceptibility between two different species or the cause may be a naturally occurring bell-curve variation in vulnerability if there are differences in susceptibility between two populations of the same species. The reduced susceptibility of the KS1 strain without prior parasiticide exposure illustrates that genetic variation within a species certainly could contribute to eventual resistance development.

Search of the Arthropod Pesticide Resistance Database (APRD) [57] at http://www.pesticideresistance.com/, which uses a qualifying RR of ≥10 to be considered resistant, revealed that for fleas of interest to veterinarians who treat dogs and cats there were 12 reports of insecticide resistance for *Ctenocephalides canis*, 28 resistance reports for *C. felis,* and 13 for *Pulex irritans.*

None of these APRD-referenced reports involve resistance to chemicals currently labeled for flea control on dogs or cats in the United States. *Ctenocephalides canis* resistance was found for BHC/cyclodienes, DDT, and HCH-gamma. *Ctenocephalides felis* resistance was found for bendiocarb, BHC/cyclodienes, carbaryl, chlordane, cyfluthrin, cypermethrin, DDT, dieldrin, fenvalerate, fluvalinate, HCH-gamma, malathion, and methoxychlor. *Pulex irritans* resistance was found for BHC/cyclodienes and DDT.

The APRD also contains reports of resistance for ticks of interest to veterinarians who treat dogs and cats. There was 1 report of acaricide resistance for *Amblyomma americanum*, 2 resistance reports for *Dermacentor variabilis,* and 9 for *Rhipicephalus sanguineus*.

*Amblyomma americanum* resistance was found for BHC/cyclodienes. *Dermacentor variabilis* resistance was found for BHC/cyclodienes and DDT. *Rhipicephalus sanguineus* resistance was found for amitraz, BHC/cyclodienes, and organophosphates. Acaricide resistance in ticks infesting dogs and cats has not been investigated as extensively as that of cattle ticks, especially *Rhipicephalus (Boophilus) microplus*, which has been intensely studied, both because of its economic importance to the cattle industry and because the species is resistant to so many compounds [58]. To provide some perspective, the APRD contains 81 reports of *Rhipicephalus microplus* resistance to the following chemicals: chlorpyrifos, cypermethrin, deltamethrin, fipronil, flumethrin, and ivermectin [57].

Regarding ticks found on dogs and cats, a strain of *Rhipicephalus sanguineus* collected in Panama was compared to susceptible strains from other areas and was classified as highly resistant to permethrin, moderately resistant to amitraz, and susceptible to fipronil [25,59]. Reports on other *Rhipicephalus sanguineus* strains suggest that resistance to deltamethrin can occur, which indicates that resistance to pyrethroid acaricides may be a concern with this tick [59]. However, studies suggest resistance varies among different populations of *Rhipicephalus sanguineus*[59]. Synergist studies indicate esterases can be involved in this tick’s resistance to pyrethroid acaricides [25].

### The concept of refugia as it applies to flea and tick resistance

Resistance development is influenced by many factors. One primary factor is the evolutionary selection pressure that a chemical puts upon an arthropod population. The portion of the arthropod population that is exposed to the chemical influences the outcome of this pressure. If the entire population is exposed, then selection pressure is increased compared to a situation where only a small portion of the population is exposed. “Refugium” is the term used when parasitologists or entomologists refer to the portion of the pest population that is not exposed to the chemical. The term is commonly used in veterinary medicine when discussing resistance of horse and ruminant helminths, but, to the authors’ knowledge, has not been used in discussions of resistance in fleas and ticks parasitizing dogs and cats. The refugia (plural of refugium) provide a reservoir of pesticide-susceptible genes because there is no selection pressure on parasites that are unexposed to the chemical(s). Management of refugia by pasture rotation and strategic administration of anthelmintics, treating only the most heavily parasitized animals, has been used in horses and ruminants to delay progression of helminth resistance.

The situation with fleas and ticks of dogs and cats is different because refugium management has not been studied or strategically used against flea and tick resistance. But an understanding of refugia can help explain differences in resistance that exist and can predict which species will be more prone to develop resistance in the future. Differences in refugia occur in various parasitic arthropods due to differences in their biology and life cycle.

Consider the cat flea. *Ctenocephalides felis* eggs, larvae, pupae, and pre-emerged adults live in the substrate around their host. While the host may be treated with insecticide, areas of the environment frequented by alternative hosts that are not exposed to insecticide provide refugia of unexposed flea eggs, larvae, pupae, and pre-emerged adults. Adult *Ctenocephalides felis* are fairly permanent ectoparasites once on a host, however this flea infests a large variety of alternative host species including coyotes, foxes, bobcats, skunks, rodents, raccoons, opossums, panthers, poultry, calves, and ferrets [4,5,42]. Cat fleas infesting untreated hosts, including feral cats, are also part of the refugium.

Consider the tick, *Rhipicephalus microplus*. This tick is resistant to more chemicals than any other [60]. *Rhipicephalus microplus* is a one-host tick. It remains on the host during two molting periods (larvae/nymph and nymph/adult) [61]. This tick primarily infests cattle. These life cycle features provide very little refugia, which made eradication possible in the United States. The only ticks unexposed to treatment were on cattle that were not treated. The eradication program was, and is, federally mandated, so essentially all tick-infested cattle in the United States were treated. The lack of refugia could be a partial explanation for the ubiquitous resistance seen in this tick species.

Consider *Rhipicephalus sanguineus* and *Amblyomma* spp. ticks. They are three-host ticks [61]. Therefore each stage (larvae, nymph, adult) must find a new host following a molt in the environment [61]. *Rhipicephalus sanguineus* prefers a dog host for each life stage [61]; which provides limited refugia for the brown dog tick, but still more than the refugia of *Rhipicephalus microplus*. This is because fed larvae and nymphs of *Rhipicephalus sanguineus* molt on the premises, are therefore not under selection pressure by topical acaricides, and once molting is completed may infest a different individual dog after each molt. *Amblyomma* spp. larvae and nymphs feed on a wide variety of species, with adult ticks found on numerous ruminants, other wild and domestic animals, and humans [61], thus providing substantial increased refugia compared to the brown dog tick. *Amblyomma maculatum* larvae and nymphs are found on a wide variety of birds, rabbits, mice, squirrels, and rats. *Amblyomma maculatum* adults have been found on domestic dogs, cats, horses, cattle, pigs, humans, and a wide variety of ruminants (deer, goats) and carnivores (bear, bobcat, panther, skunk, raccoon, fox, coyote) [62]. This life cycle provides vast refugia for *Amblyomma* spp., and other 3-host ticks such as *Dermacentor* spp. and *Ixodes* spp., and therefore much less selection pressure for resistance development by these species compared to the brown dog tick. Thus in any given questionable tick efficacy situation, identification of tick species is helpful because while treatment deficiency is most likely causal, suspicion of brown dog tick resistance will be more credible than resistance of any of the other ticks species that infest dogs and cats.

Refugia management (avoiding chemical administration to a proportion of susceptible individuals) is one strategy that has been employed to reduce future resistance [17,56], but one that is not employed by veterinary practitioners when dealing with flea and tick infestations because it is impractical and is likely unnecessary when dealing with pests with large refugia [63].

### Acaricide and insecticide alternatives

Several potential flea or tick pathogens have been proposed as biological parasite control agents. Such strategies for controlling pest populations and managing resistance have been employed in other areas of entomology. However, to date similar alternatives have not been very successful with fleas and ticks. Entomopathogenic (organisms that kill arthropods) nematodes, such as *Neoaplectana carpocapsae*[64] and *Steinernema carpocapsae*[63,65], and fungi, such as *Beauveria bassiana*[66], have been studied. *Steinernema carpocapsae* is commercially available, is marketed as effective against fleas, and could be considered if its use was practical and proven efficacious. This nematode must be applied to soil that is moist (≥20% moisture), among other things, which limits its practicality and efficacy, particularly since the soil moisture content that best suits cat flea larvae development is 1 – 10% [63,65,67]. Vaccination of dogs and cats against fleas or ticks may be possible in the future, but is not a current option [5,68-70].

### Strategies to minimize the development, progression, and impact of resistance

The use of a program that targets both adult and environmental flea life stages may decrease the rate of resistance development [5,71]. Such an approach may involve the use of insect growth regulators (juvenile hormone analogs or chitin synthesis inhibitors), ovicides, adulticides, and physical or mechanical intervention. Practitioners should consider investigating the mode of action of chemical agents currently used against fleas and/or ticks in premises or on dogs and/or cats when developing their program [38,43,72-74]. Development of such a program is a commonly used strategy by veterinarians who provide an integrated management system that includes educating veterinary staff and pet owners on flea biology, instructing owners on proper use of mechanical control systems (such as vacuuming, washing pet bedding, and the use of light traps), dispensing products that provide effective flea adulticide and environmental life stage control, and promoting realistic owner expectations [63].

Bathing and swimming may reduce insecticide and acaricide levels of some topically applied products [7,63]. No product can kill or repel every flea or tick immediately and it is unlikely that these products will retain 100% efficacy throughout the labeled duration of activity. Therefore, when dogs and cats are exposed to overwhelming populations of fleas or ticks, owners may continue to see fleas and ticks, even if the products are performing as labeled. Seeing moving, but dying fleas for 1–3 months after instituting topical monthly adulticide therapy should be expected in these cases. When investigating resistance it is important to rule out product failure that occurs because of incorrect storage, dilution, application, or unusual climactic or environmental conditions [60]. The most common reasons found for explaining pet owner lack of efficacy reports relate to inconsistent treatment with insecticides and acaricides (failure administer the product at correct intervals or to administer the product at all) or continued parasite exposure, the latter of which is as a result of the presence of infested wildlife in the case of fleas or incomplete treatment of the premises or environment in the case of both fleas and ticks.

Regardless of the reason for the apparent lack of efficacy it is important to contact manufacturers regarding use of their products, especially if resistance is suspected. The technical service department may have helpful suggestions on how to work the case up with the pet owner and document the situation accurately. Manufacturers report all complaints and lack of efficacy calls to the appropriate government agency.

More studies are needed. Investigating true resistance and determining that it exists for a particular population of parasites with a particular insecticide/acaricide is not an easy process; it takes time and costs money. The ultimate responsibility of the veterinary practitioner is to provide pets with relief from flea and tick infestations and keep animal owners satisfied. If there are questions regarding the efficacy of a particular treatment, and this treatment is an adulticide, then the practitioner may conduct a basic test for treatment susceptibility by applying the product in the office, housing the infested patient in a controlled area for sufficient time, and then checking for adult parasites (if confident that newly emergent fleas will not jump onto the patient in the clinic). This type of clinical impression test does not provide an accurate measure of resistance, but can provide a relative estimate of efficacy if the same process is repeated with an alternate product. If far fewer infesting parasites are seen at the end of the appropriate period for the alternate product, then why not switch? When testing an insecticide in-clinic using an evaluation such as described above, one must be careful interpreting the results. This in-clinic test may not accurately reflect how the product will perform in the home because the product’s full range of activity will not be measured. Some products rely heavily on ovicidal or other types of non-adulticide activity, which may not be assessed by this test. It certainly should not be used to condemn a particular insecticide, given that such an evaluation is basically an n of 1. The result of an experiment with only one test subject and no control group is definitely not solid scientific evidence. While lack of efficacy might be due to resistance, it may also be caused by the way the product distributes on or is absorbed by the individual animal or may be due to innate reduced susceptibility. But clinically, regardless of the reason, a switch may be necessary to protect the health of this individual pet and provide client satisfaction. It is important in each case to review the patient history looking for possible treatment program deficiencies.

## Conclusions

When lack of insecticide or acaricide efficacy is noted by a veterinary practitioner or reported by the pet owner, it is essential to review the history and look for potential treatment deficiency, because the ultimate cause is much less likely to be actual flea or tick resistance. If reduced susceptibility to treatment is seen, then other more common causes must be ruled out before resistance can be considered as likely. Resistance to pesticide treatment only becomes an accurate diagnosis when it can be shown that the parasite population has changed as a consequence of selection pressure created by past exposure to a specific insecticide. With today’s situation regarding finding proof of resistance, a practitioner’s opinion of the cause of the efficacy problem will ultimately be anecdotal rather than proven unless they just happen to find a manufacturer or academic researcher pursuing a resistance study. Regardless of the cause, perceived lack of efficacy may require a revised treatment approach to satisfy the owner and the veterinarian.

## Abbreviations

AChE: Acetylcholinesterase; APRD: Arthropod pesticide resistance database; BHC: Benzene hexachloride; DDT: Dichlorodiphenyltrichloroethane; EPA: United States environmental protection agency; FAO: Food and agriculture organization; FDA: United States food and drug administration; GABA: Gamma-aminobutyric acid; HCH: Hexachlorocyclohexane; Kdr: Knockdown resistance; LIM: Larval immersion microassay; LPT: Larval packet test; nAChR: Nicotinic acetylcholine receptor; PCR: Polymerase chain reaction; Rdl: Resistance to dieldrin gene; RR: Resistance ratio; WHO: World Health Organization.

## Competing interests

TBC has worked for numerous pharmaceutical companies and been acknowledged for contributing writing, editing, and literature search for many clinical trial reports and marketing documents.

MWD has had research funded and has been sponsored to lecture by numerous pharmaceutical companies, including Merck Animal Health.

## Authors’ contributions

TBC proposed the review article to MWD and provided the outline, which MWD edited. TBC performed the majority of the literature search, writing, and editing. MWD provided direction, editing, and advanced expertise in the topic area. Both authors agreed upon the text submitted to the publisher and approved the final version of the manuscript.
